# Tissierella praeacuta Bacteremia Associated With Acute on Chronic Osteomyelitis: A Case Report

**DOI:** 10.7759/cureus.44962

**Published:** 2023-09-09

**Authors:** Amteshwar Singh, Nassar Patni, Ashampreet Kaur, Amitasha Sinha, Waseem Khaliq

**Affiliations:** 1 Medicine, Johns Hopkins Bayview Medical Center, Baltimore, USA; 2 Internal Medicine, Johns Hopkins University School of Medicine, Baltimore, USA; 3 Internal Medicine, Deccan College of Medical Sciences, Hyderabad, IND; 4 Internal Medicine, Sri Guru Ram Das Institute of Medical Sciences and Research, Amritsar, IND; 5 Hospital Medicine, Beth Israel Deaconess Medical Center, Harvard Medical School, Boston, USA; 6 Medicine, Johns Hopkins University School of Medicine, Baltimore, USA

**Keywords:** tissierella, ivdu, bacteremia, cellulitis, osteomyelitis

## Abstract

*Tissierella praeacuta* is a rare gram-variable bacillus that naturally occurs in the environment and is pathogenic in humans with chronic infections. We report the case of a 45-year-old man with a history of chronic osteomyelitis of the left tibia and recurrent bacteremia secondary to intravenous drug use (IVDU). He had previously received multiple partially completed courses of antibiotics over the past one year. Blood cultures demonstrated polymicrobial infection, including *T. praeacuta *and methicillin-sensitive *Staphylococcus aureus* managed with parenteral beta-lactams, and the subsequent first surveillance cultures remained sterile. Medical literature on human infections with *T. praeacuta *is limited due to its rare occurrence. Most cases have reported sensitivity to beta-lactam antibiotics, making them an antibiotic of choice. *T. praeacuta* infections should prompt a search for additional underlying infectious foci and treatment of any additional co-infecting microbes.

## Introduction

Tissierella praeacuta is a gram-variable bacillus that naturally occurs in the soil and has been rarely reported as pathogenic in humans [1,2]. It has reportedly been a causal agent in polymicrobial infections. We report the case of a 45-year-old man with active intravenous drug use (IVDU) who was found to have T. praeacuta bacteremia in the setting of acute on chronic tibial osteomyelitis. A literature search of case reports using the terms "Tissierella praeacuta” or “Clostridium hastiforme” in the PubMed/MEDLINE database did not reveal any other cases of T. praeacuta infection in a patient with IVDU.

## Case presentation

**Figure 1:**
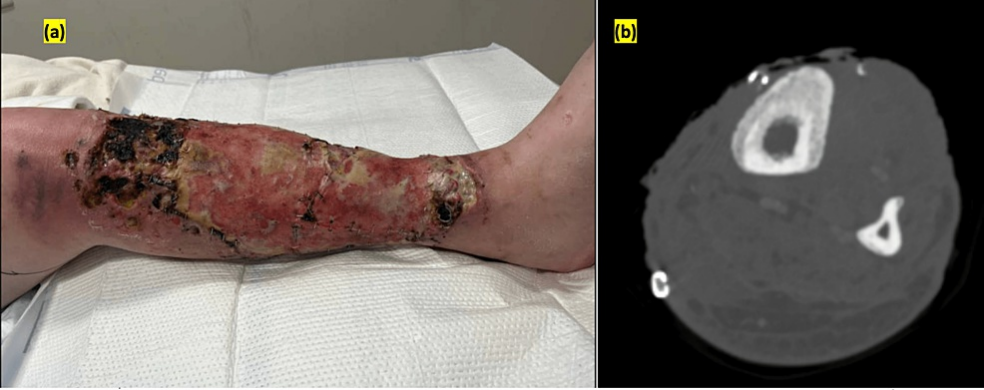
(a) Image of the left lower extremity cellulitis. (b) Contrast-enhanced computed tomography scan of the left lower extremity showing an underlying periosteal reaction, erosive changes, and cortical thickening along the left tibial shaft, suggestive of osteomyelitis.

A 45-year-old man with a history of chronic hepatitis C infection, opioid IVDU, chronic osteomyelitis of bilateral lower extremities, and right below-knee amputation secondary to osteomyelitis presented to the hospital with fever and progressive worsening of left leg pain associated with purulent discharge from chronic left leg wound for four days. His left leg wound developed four months ago at an IVDU injection site that gradually increased in size and became purulent. He had been previously treated on multiple occasions over the past four months for recurrent IVDU-associated cellulitis and bacteremia (Bacillus cereus, carbapenem-resistant Acinetobacter baumannii, Streptococcus viridins, Staphylococcus aureus, Staphylococcus hominis, Clostridium sporogenes, Clostridium botulinum, and Pseudomonas aeruginosa) with intravenous vancomycin, cefepime, metronidazole, and oral trimethoprim-sulfamethoxazole. He had a long history of prematurely leaving against medical advice during hospitalizations after partial antibiotic treatments, including his last hospitalization when he also underwent debridement and skin graft placement one month ago. Despite extensive counseling from inpatient substance use recovery service, he reported continued struggle with intravenous drug injection into the leg wound. The patient reported housing instability. In addition, while he didn’t report any overt contamination of the wound with soil, he admitted to suboptimal hygiene and wound care prior to the hospitalization. In the current presentation, his vitals were pertinent for hypothermia of 35.7°C and tachycardia of 116 beats per minute. Local examination revealed an ulcerated wound (32x24 cm) with multiple staples from previous skin graft surgery on the left leg with erythema, swelling, and tenderness (Figure 1a). Laboratory evaluation showed normal leukocyte count, microcytic anemia (hemoglobin 10.1 g/dl), elevated C- reactive protein of 5.74 mg/dl (Ref range <0.29 mg/dl), and erythrocyte sedimentation rate of 42 mm/hour (Ref range <=15 mm/hour). The human immunodeficiency virus (HIV) test was negative. Left leg computed tomography showed periosteal thickening and erosions in the mid-to-distal tibia and fibula, suggesting chronic osteomyelitis (Figure 1b).

Empiric parenteral vancomycin, ceftriaxone, and metronidazole were started. On day 2, blood cultures grew gram-variable anaerobic bacilli, confirmed as T. praeacuta (sensitive to beta-lactams, clindamycin, and metronidazole); therefore, antibiotic coverage was switched to intravenous ampicillin-sulbactam on day 3. Wound debridement was performed on day 6. Intraoperative tissue cultures grew methicillin-sensitive Staphylococcus aureus, Enterobacter cloacae, Enterococcus faecalis, and Clostridium sporogenes. Oral ciprofloxacin was added to ampicillin-sulbactam to treat the resistant Enterobacter strain. On day 13, the patient decided to leave prematurely against medical advice. He was educated to complete oral ciprofloxacin and doxycycline course of 6 weeks, abstinence from IVDU, and clinic follow-up with infectious disease. Notably, the patient was followed by the addiction medicine consult service as well as peer recovery coach services throughout his stay. He was provided extensive abstinence counseling and given resources for outpatient follow-up with the addiction medicine team.

## Discussion

T. praeacuta is a gram-variable obligate-anaerobic bacillus, originally named Bacteroides praeacutus by H. Tissierella after extraction from infant feces in 1908 [1]. Clostridium hastiforme, a closely related bacterium, is now renamed Tissierella praeacuta, classified under phylum Firmicutes, order Clostridiales, and family Peptostreptococcaceae (or incertae sedis XI) [1,2]. T. praeacuta naturally occurs in anaerobic sludge, soil, and human microbiota. Of the five species under the Tissierella genus (T. praeacuta, T. creatinini, T.pigra, T. carlieri, and T. creatinophila), only T. praeacuta is reported to be pathogenic to humans [3]. A literature search of case reports using the terms "Tissierella praeacuta” or “Clostridium hastiforme” in the PubMed/MEDLINE database did not reveal any other cases of T. praeacuta infection in a patient with IVDU. Tissierella bacteremia has been previously reported to co-infect patients with chronic infections such as calcaneal osteomyelitis in a diabetic patient [4] and sacral decubitus ulcer [3], successfully treated with beta-lactams. Other reported cases include knee arthroplasty, pyonephrosis with communicating liver abscess [5], brain abscess secondary to otitis media in a child [6], sepsis secondary to colon adenocarcinoma [7], eyelid gas gangrene [8], and pyometra secondary to an intrauterine device [9]. Most cases are successfully treated with beta-lactams, although susceptibility to rifampicin and chloramphenicol is also reported.

We performed a literature search of the case reports/series published in the English language from inception to August 2023 using the terms "Tissierella praeacuta” or “Clostridium hastiforme” in the PubMed/MEDLINE database. A supplementary search was carried out by two authors (AS and WK) manually to locate any additional publications. This yielded a total of 10 known cases of pathogenic and one colonizer, Tissierella praeacuta (Table 1).

**Table 1:** A literature review of reported cases of Tissierella praeacuta. M- male; F- Female.

AUTHOR	YEAR	Age, Sex	DIAGNOSIS	CO-PATHOGEN	SUSCEPTIBILITY	TREATMENT
Yang et al. [3]	2022	45, M	Chronic Sacral Wounds	Proteus mirabilis, Prevotella bergensis, Bacteroides fragilis, and Parvimonas sp.	Not reported	Piperacillin-tazobactam 3.375 g every six hours IV for a total of 14 days
Gill et al. [4]	2022	62, M	Osteomyelitis	Proteus penneri/vulgaris and Enterococcus faecalis.	Not reported	Wound debridement and vacuum-assisted closure. Initial treatment with cefepime and metronidazole. Additional six-week treatment with oral Levofloxacin and Amoxicillin-Clavulanate.
Caméléna et al. [5]	2016	35, M	Pseudarthrosis of fractured long bone (femur)	Enterobacter cloacae.	Piperacillin – tazobactam Beta lactams Metronidazole Chloramphenicol Rifampicin	Piperacillin/tazobactam 4 g three times a day IV and metronidazole 500 mg three times a day for six weeks.
Caméléna et al. [5]	2016	74, M	Pyonephrosis	Streptococcus anginosus, Proteus mirabilis and an extended- spectrum beta-lactamase-producing Escherichia coli.	Piperacillin – tazobactam Metronidazole Chloramphenicol Rifampicin	Meropenem 3 g per day for 14 days
Cox et al. [6]	2009	10, M	Brain abscess Otitis media	Morganella morganii, Proteus mirabilis, Corynebacterium amycolatum, Bacteroides fragilis, and Enterobacter cloacae.	Not reported	Meropenem 120 mg/kg/day every eight hours failed percutaneous drainage Abscess resection with craniotomy
Houssany et al. [7]	2016	67, F	Septic shock Colorectal cancer with multiple stomas. Chemotherapy-induced aplasia.	Escherichia coli and Staphylococcus aureus.	Piperacillin Amoxicillin/clavulanate Cefotaxime Imipenem Chloramphenicol Clindamycin Ciprofloxacin Metronidazole Vancomycin.	Piperacillin – tazobactam for 14 days
Lyon et al. [8]	1989	31, M	Eyelid Gas gangrene	None.	Metronidazole Ceftriaxone Penicillin	Cephalexin 500 mg four times daily Surgical debridement
Ørum et al. [9]	2017	64, F	Pyometra	Bacteroides fragilis.	Penicillin G Meropenem Clindamycin Metronidazole	Cefuroxime intravenous 1500 mg and metronidazole 500 mg, both three times a day for six days. Oral pivampicillin 700 mg three times a day and metronidazole 500 mg twice a day for an additional 7 days.
Williamson et al. [10]	1977	4, M	Colonizer in Severe combined immune deficiency (SCID)	About 35 microorganisms (unknown if contaminants).	Not reported	Not treated. Reportedly colonizer. Source of specimen not reported.
Welsh et al. [11].	2015	49, F	Leg wound	None.	Not reported	Piperacillin – tazobactam
Samanta et al. [12]	2016	49, F	Rectal carcinoma Rectovaginal fistula Intrauterine device (IUD) present	None.	Not reported	Explorative laparotomy, palliative diverting loop colostomy, IUD removal. Piperacillin – tazobactam, then Metronidazole for a total antibiotic course of 14 days.
Chandok et al. [13]	2023	24, F	Septic Ovarian Thrombophlebitis	None.	Beta-lactams Chloramphenicol Meropenem Metronidazole	Piperacillin-tazobactam was switch to meropenem. Discharged on oral metronidazole with oral amoxicillin-clavulanate.

For patients with bacteremia-related osteomyelitis and IVDU, the possibility of rare pathogens like T. praeacuta should be suspected, especially when the clinical course is prolonged, or bacteremia is polymicrobial.

## Conclusions

Very little is known about the prevalence and risk factors associated with T. praeacuta due to scant reporting in the current literature. T. praeacuta generally occurs as a co-infecting pathogen and should be considered in the differentials, especially for patients suspected to be at-risk, like IVDU or with chronic infections. This is the first case description of T. Praeacuta infection in a patient using drugs and actively injecting into his wound. T praeacuta is generally sensitive to beta-lactam antibiotics; a comprehensive treatment plan entails source identification, antibiotic treatment with beta-lactams, and/or surgical intervention if indicated. 
